# Robust Brain Hyperglycemia during General Anesthesia: Relationships with Metabolic Brain Inhibition and Vasodilation

**DOI:** 10.3389/fphys.2016.00039

**Published:** 2016-02-15

**Authors:** R. Aaron Bola, Eugene A. Kiyatkin

**Affiliations:** In-Vivo Electrophysiology Unit, Behavioral Neuroscience Branch, National Institute on Drug Abuse – Intramural Research Program, National Institutes of HealthBaltimore, MD, USA

**Keywords:** brain and body hypothermia, metabolic brain inhibition, anesthesia, vasoconstriction, vasodilation, nucleus accumbens, rats

## Abstract

Glucose is the main energetic substrate for the metabolic activity of brain cells and its proper delivery into the extracellular space is essential for maintaining normal neural functions. Under physiological conditions, glucose continuously enters the extracellular space from arterial blood via gradient-dependent facilitated diffusion governed by the GLUT-1 transporters. Due to this gradient-dependent mechanism, glucose levels rise in the brain after consumption of glucose-containing foods and drinks. Glucose entry is also accelerated due to local neuronal activation and neuro-vascular coupling, resulting in transient hyperglycemia to prevent any metabolic deficit. Here, we explored another mechanism that is activated during general anesthesia and results in significant brain hyperglycemia. By using enzyme-based glucose biosensors we demonstrate that glucose levels in the nucleus accumbens (NAc) strongly increase after iv injection of Equthesin, a mixture of chloral hydrate and sodium pentobarbital, which is often used for general anesthesia in rats. By combining electrochemical recordings with brain, muscle, and skin temperature monitoring, we show that the gradual increase in brain glucose occurring during the development of general anesthesia tightly correlate with decreases in brain-muscle temperature differentials, suggesting that this rise in glucose is related to metabolic inhibition. While the decreased consumption of glucose by brain cells could contribute to the development of hyperglycemia, an exceptionally strong positive correlation (*r* = 0.99) between glucose rise and increases in skin-muscle temperature differentials was also found, suggesting the strong vasodilation of cerebral vessels as the primary mechanism for accelerated entry of glucose into brain tissue. Our present data could explain drastic differences in basal glucose levels found in awake and anesthetized animal preparations. They also suggest that glucose entry into brain tissue could be strongly modulated by pharmacological drugs via drug-induced changes in metabolic activity and the tone of cerebral vessels.

## Introduction

Glucose is the main energetic substrate for the metabolic activity of brain cells (Siesjo, 1978; Sokoloff, 1999; Mergenthaler et al., 2013) and its proper delivery into the extracellular space is essential for maintaining normal neural functions. Unlike most neurochemicals that are locally synthetized and released either with the synapse or into the extracellular space due to neural activation, glucose enters the brain tissue from the arterial blood via gradient-dependent facilitated diffusion via the GLUT-1 glucose transporter (Duelli and Kuschinsky, 2001). Under physiological conditions, glucose entry into brain tissue is governed by two primary mechanisms. First, glucose levels in the extracellular space increase due to global rises in blood glucose concentrations (de Vries et al., 2003; Dunn-Meynell et al., 2009; Dash et al., 2013). Such “passive” increases occur during consumption of glucose-containing products and intra-gastric glucose or systemic glucose injections, and they are slow and relatively large, directly depending upon the amount of ingested or injected glucose (Wakabayashi and Kiyatkin, 2015a). In addition to this slow, gradient-dependent mechanism, glucose could rapidly enter the brain due to increases in local blood flow resulting from proximal neural activation (Fellows et al., 1992; Silver and Erecinska, 1994; Attwell et al., 2010). These increases are much more rapid, but less in magnitude (20–100 μM) and more transient (Kiyatkin and Lenoir, 2012).

Here, we provide evidence for another mechanism that is activated during general anesthesia and determines robust entry of glucose into brain tissue due to vasodilation of cerebral vessels. First, by using enzyme-based glucose biosensors coupled with amperometry, we demonstrate that the development of general anesthesia results in a rapid, robust, and prolonged rise in extracellular glucose levels in the nucleus accumbens shell (NAc). Second, by simultaneous temperature recordings from the NAc, temporal muscle, and skin, we show that general anesthesia results in robust decreases in brain and body temperatures and clarify its two underlying mechanisms: decrease in intra-brain heat production due to metabolic inhibition and increase in heat loss due to vasodilation. Finally, by using correlation and regression analyses, we show a tight correlation between increases in NAc glucose levels and Skin-Muscle temperature differentials, a valid measure of peripheral vasodilation (Kiyatkin, 2010). We chose the NAc, a critical structure in integrating sensory and motor processes for behavioral output (Mogenson et al., 1980; Wise and Bozarth, 1987), as a representative deep brain structure for our electrochemical and temperature recordings. Based on these data, we assume that the robust rise in brain glucose occurring during general anesthesia results from powerful dilation of cerebral vessels, which enhances glucose entry from the arterial blood into the brain's extracellular space. The effect of anesthesia could explain the existing controversy regarding basal glucose levels in brain tissue determined in awake and anesthetized animal preparations (see McNay et al., 2001; Routh, 2002 for details) and question the validity of findings on fluctuations in extracellular glucose obtained in anesthetized animals. The knowledge on true concentrations of brain glucose under physiologically relevant conditions is also important for understanding of glucose sensing by brain cells and for providing optimal conditions for *in vitro* assessment of neural activity.

## Materials and methods

### Animals and housing

Forty-five male Long-Evans rats (440 ± 40 g) supplied by Charles River Laboratories (Greensboro, NC) were housed individually in a temperature-, humidity-, and light-controlled room (12/12 h light/dark cycle, lights on at 07:00) with free access to food and water. Protocols were performed in compliance with the Guide for the Care and Use of Laboratory Animals (NIH, Publication 865-23) and were approved by the NIDA-IRP Animal Care and Use Committee.

### General structure of the study

Our previous experiments using enzyme-based glucose biosensors were conducted in awake, freely moving rats and in most cases animals were equipped with intravenous (iv) catheter. Since post-recording sensor calibration is an essential part of our experimental paradigm, at the final stage of these experiments rats were injected with 0.8–0.9 ml of Equithesin to induce general anesthesia and remove safely the sensor for calibration. Equithesin is a general anesthetic preparation that is often used during surgeries in rats; its active ingredients are sodium pentobarbital and chloral hydrate, (9.7 and 44.4 mg/ml, respectively). Since the analgesic and behavior-blocking effects of this treatment occur at the end of the injection, our electrochemical recordings continued for at least 8 min. Data obtained during these recordings composed the first data set of the present study.

The robust changes in NAc glucose we consistently observed in these experiments led us to conduct a second experiment, in which we examined the effects of Equithesin administered at the same dose, route of administration, and injection speed on NAc glucose levels during long-term recording. In this experiment, we also conducted several control tests to examine the possible mechanisms underlying the observed dynamics of glucose fluctuations. This experiment produced the second data set of the present study.

To further understand the possible mechanisms underlying robust anesthesia-induced changes in glucose, we conducted the third experiment, in which we examined temperature changes in NAc, temporal muscle, and skin induced by iv injections of Equithesin used at the same dose and administration route. While it is known that general anesthesia induces hypothermia and we previously examined this effect using intraperitoneal (ip) injections of sodium pentobarbital (50 mg/kg; Kiyatkin and Brown, 2005), the three-point recording paradigm allowed us to examine the dynamics of two primary variables underlying the hypothermic effects of anesthesia: intra-brain heat production and vascular tone. These data were used to conduct correlative and regression analyses aimed to examine the possible mechanisms responsible for specific changes in glucose found in our electrochemical experiments.

### Common procedures in all experiments

In all experiments, rats were implanted with a chronic jugular catheter, which ran subcutaneously to a head mount constructed specifically for either experiment (see below), made from dental acrylic and secured to the skull by three stainless steel bone screws. Rats were allowed a minimum of 4 days of post-operative recovery; jugular catheters were flushed daily with 0.2 ml heparinized saline (10 units/ml) to maintain patency. At the onset of each experiment, the injection port of the jugular catheter on the head mount was connected to plastic catheter extensions that allowed stress- and cue-free delivery of tested substances from outside the chamber, thus minimizing possible detection of the injection procedure by the rat.

All rats underwent similar habituation to the testing environment (a minimum of 6 h a day for 3 consecutive days) prior to the recording sessions. Recording experiments began 5 days after surgery and were conducted during the light-phase of the cycle (10:00–17:00). All recordings were conducted in standard chambers (38 × 47 × 47 cm) under continuous dim illumination (20 W red light bulb), with a room wide air filter fan providing background noise.

In each experiment, rats received either one or two iv injections of Equithesin (0.8–0.9 ml depending on animal weight or 18 mg/kg for sodium pentobarbital plus 84 mg/kg for chloral hydrate), which was delivered at the same speed and duration (120 s) with minimal possible stressful or arousing influence of the injection procedure.

### Electrochemical experiments (I and II)

#### Surgical preparations

Each rat used in these experiments was surgically prepared for electrochemical recordings as described in detail previously (Kiyatkin and Lenoir, 2012; Wakabayashi and Kiyatkin, 2014, 2015a). Briefly, under general anesthesia (Equithesin 0.33 ml/100 g, ip), rats were implanted with a BASi cannula (Bioanalytical Systems, Inc.; West Lafayette, IN, USA) for future insertions of a biosensor in the medial sector of the nucleus accumbens (NAc shell). The target coordinates were: AP +1.2 mm, ML ± 0.8 mm and DV 7.3 mm, according to the stereotaxic atlas of Paxinos and Watson (1998). The guide cannula hub was fixed to the skull with a head mount constructed from dental acrylic that was secured using three stainless steel bone screws.

#### Fixed-potential amperometry with enzyme-based glucose sensors

Commercially produced glucose oxidase-based biosensors (Pinnacle Technology, Inc., Lawrence, KS, USA) coupled with fixed-potential amperometry have been extensively used in our previous studies (Kiyatkin and Lenoir, 2012; Kiyatkin et al., 2013; Kiyatkin and Wakabayashi, 2015; Wakabayashi and Kiyatkin, 2015a,b). These reports describe in detail multiple issues regarding the sensitivity/selectivity of these sensors, their *in vitro* and *in vivo* performance, and possible physical and chemical contributions that could be evaluated and controlled for providing high reliability and accuracy of electrochemical measurements of extracellular glucose fluctuations.

Briefly, glucose sensors are prepared from Pt-Ir wire of 180 μm diameter, with a sensing cavity of ~1 mm length on its tip. The active electrode is incorporated with an integrated Ag/AgCl reference electrode. On the active surface, glucose oxidase converts glucose to glucono-1,5-lactone and hydrogen peroxide (H_2_O_2_), which is detected as an amperometric oxidation current generated by a +0.6 V applied potential (Hu and Wilson, 1997). The potential contribution of ascorbic acid to the measured current is competitively reduced by co-localizing ascorbic acid oxidase enzymes on the active surface of the sensor. This enzyme converts ascorbic acid to non-electroactive dehydroascorbate and water. In addition, a negatively charged Nafion polymer layer under the enzyme layer serves to exclude endogenous anionic compounds (Hu and Wilson, 1997).

Glucose sensors were calibrated immediately before and after each *in vivo* experiment. *In vitro* calibrations were conducted in PBS (pH 7.3) at room temperature (23°C) by incrementally increasing the concentration of glucose (Sigma-Aldrich) from 0 to 0.5, 1.0, and 1.5 mM followed by a single addition of ascorbate (25 or 250 μM). Within the physiological range of glucose levels (Fellows and Boutelle, 1993; McNay et al., 2001), glucose sensors produced incremental linear current increases, with a mean sensitivity of 3.12 ± 0.27 nA/0.5 mM. Glucose sensors showed low sensitivity to ascorbate (0.17 ± 0.06 nA/25 μM) and, as showed previously, they were only minimally sensitive to dopamine at its physiological levels (5-50 pA/10–100 nM). As shown previously (see Kiyatkin and Wakabayashi, 2015), glucose sensors remained equally sensitive to glucose and selective against ascorbate during post-recording *in vitro* calibrations. Since the sensitivity of electrochemical sensors is greatly affected by temperature, our calibration data were adjusted to 37°C (6.12 ± 0.52 nA/0.5 mM) based on a temperature coefficient previously determined in analytical *in vitro* tests. Although our previous data suggest that both physical and chemical contributions to glucose electrochemical currents are minimal, we also conducted the same tests using enzyme-free or null sensors. Data obtained with these sensors were used for more precise determination of basal levels of glucose and its fluctuations in terms of concentration.

#### Protocol

At the beginning of each experimental session in Experiments I and II, rats were minimally anesthetized (<2 min) with isoflurane and a calibrated glucose or null sensor was inserted into the brain through the guide cannula. The sensor was connected to the potentiostat (Model 3104, Pinnacle Technology) via an electrically shielded flexible cable and a multi-channel electrical swivel. Additionally, the injection port of the jugular catheter on the head mount was connected to a plastic catheter extension that allowed stress- and cue-free drug delivery from outside the chamber.

The first set of electrochemical data (Experiment I) was obtained during analyses of our previous experiments obtained in awake, freely moving rats that received a single iv injection of Equithesin as the final experimental event. The injection was conducted when the rat was in quiet resting conditions ~7–8 h after the start of continuous recording. In Experiment II, rats received two iv injections of Equithesin with an inter-injection interval of at least 120 min. In this experiment, we also used one or two tests with a novel object to verify the sensor's performance and observe the pattern of NAc glucose responses induced by arousing sensory stimulation. A small 50-ml glass beaker was introduced manually to the chamber and removed 60 s thereafter. While the anesthetic drug in Experiment I was delivered at room temperature, in Experiment II we also compared the effects of anesthetic drug delivered at 23 and 37°C. For the latter test, the injection syringe was warmed in a water bath with strict temperature monitoring. In this experiment, we also examined the effects of saline delivered at room temperature at the volume (0.85 ml) identical to that used for Equithesin.

#### Data processing and statistical analyses for experiments I and II

Electrochemical data were sampled at 1 Hz (i.e., mean current over 1 s) using the PAL software (Version 1.5.0, Pinnacle Technology) and analyzed using different time resolutions. Slow changes in electrochemical currents were analyzed with 2-min quantification bins using an analysis window of 20 min before and 120 min after each iv injection. Rapid current changes were analyzed with 2 and 10-s bins for specified durations after the onset of each experimental event. Since the baseline currents slightly varied in amplitude between individual glucose electrodes, absolute current changes were transformed into relative changes by taking a basal value before each event as 0 nA. These current changes were then transformed into glucose concentration (μM) based on the sensor sensitivity determined during pre-recording *in vitro* calibrations and adjusted by the temperature coefficient (95.6%) determined in previous analytical tests (Kiyatkin et al., 2013).

Statistical data analyses included the use of one-way repeated measure (RM) ANOVAs to find time periods where there was a significant post-injection main effect. Fisher *post-hoc* tests were used for pair-wise comparisons, and the latency of the glucose response was determined based on the first data point significantly different from baseline (*p* < 0.05).

### Thermorecording experiment (III)

#### Surgical preparations

Rats for this experiment underwent the three-point thermocouple electrode implantation procedure described in detail elsewhere (Kiyatkin et al., 2014). Briefly, under general anesthesia, we implanted miniature copper-constantan thermocouple probes (125 μm in diameter) in the NAc shell (AP +1.2 mm; L 0.9 mm; DV 7.2–7.4 mm), deep temporal muscle, and subcutaneously along the nasal ridge with the tip ~15 mm anterior to bregma. Testing chambers were equipped with an electrical swivel and a flexible cable that attached to the implanted thermoelectrodes during recording sessions.

#### Protocol

The experimental protocol was similar to that in Experiment II. After ~2-h habituation to the recording chamber, rats were exposed to the novelty test (a 50-ml glass beaker presented for 60 s) and received a single injection of Equithesin 60 min after. In contrast to electrochemical recordings, when each rat was tested during only one session, temperature recordings in each rat were conducted during two sessions with one free day between them.

#### Temperature data analyses

Temperature data were continuously recorded at a 10-s resolution using Thermes-16 (Physitemp Instruments). These primary data were then analyzed at different time resolutions as absolute changes, as changes relative to the immediate pre-injection baseline values, and as NAc-Muscle and Skin-Muscle temperature differentials (i.e., the difference between relative temperature changes in the corresponding locations). Slow changes in both temperature and locomotion were analyzed in 2-min quantification bins using an analysis window of 20 min before and 120 min after each injection. Rapid changes were analyzed in 10-s bins for 60 s before and 480 s after each iv drug injection.

The brain and the temporal muscle receive arterial blood from the same carotid artery and thus are equally exposed to blood-delivered heat from the body. The NAc-Muscle temperature differential excludes this common source of heat and provides a measure of drug-induced metabolic brain activation or inhibition. Skin temperature is determined by the state of peripheral vessels, but also depends on the temperature of arterial blood inflow. Therefore, Skin-Muscle temperature differentials exclude the contribution of the latter and serve as an accurate measure of peripheral vascular tone (Kiyatkin, 2010).

Temperature data were statistically analyzed by using one-way RM ANOVAs to find time periods where there was a significant post-injection main effect and individual bins were compared with respect to baseline using Fisher *post-hoc* tests. To examine the relationships between temperature and neurochemical parameters, we also used correlation and regression analyses.

## Results

### Basal levels of NAc glucose and its changes associated with the initiation of general anesthesia

Our previous estimates of basal extracellular glucose levels in the NAc ranged from 540 μM (Kiyatkin and Lenoir, 2012) to 664 μM (Wakabayashi et al., 2015). These values were determined in quietly resting rats before the first experimental event of each study, ~2.5-h after the start of *in vivo* recording. We also previously reported that basal glucose levels became slightly lower (~80% of the initial baseline) when determined before the last experimental event, ~7.5 h after the start of *in vivo* recording.

**Figure 1:**
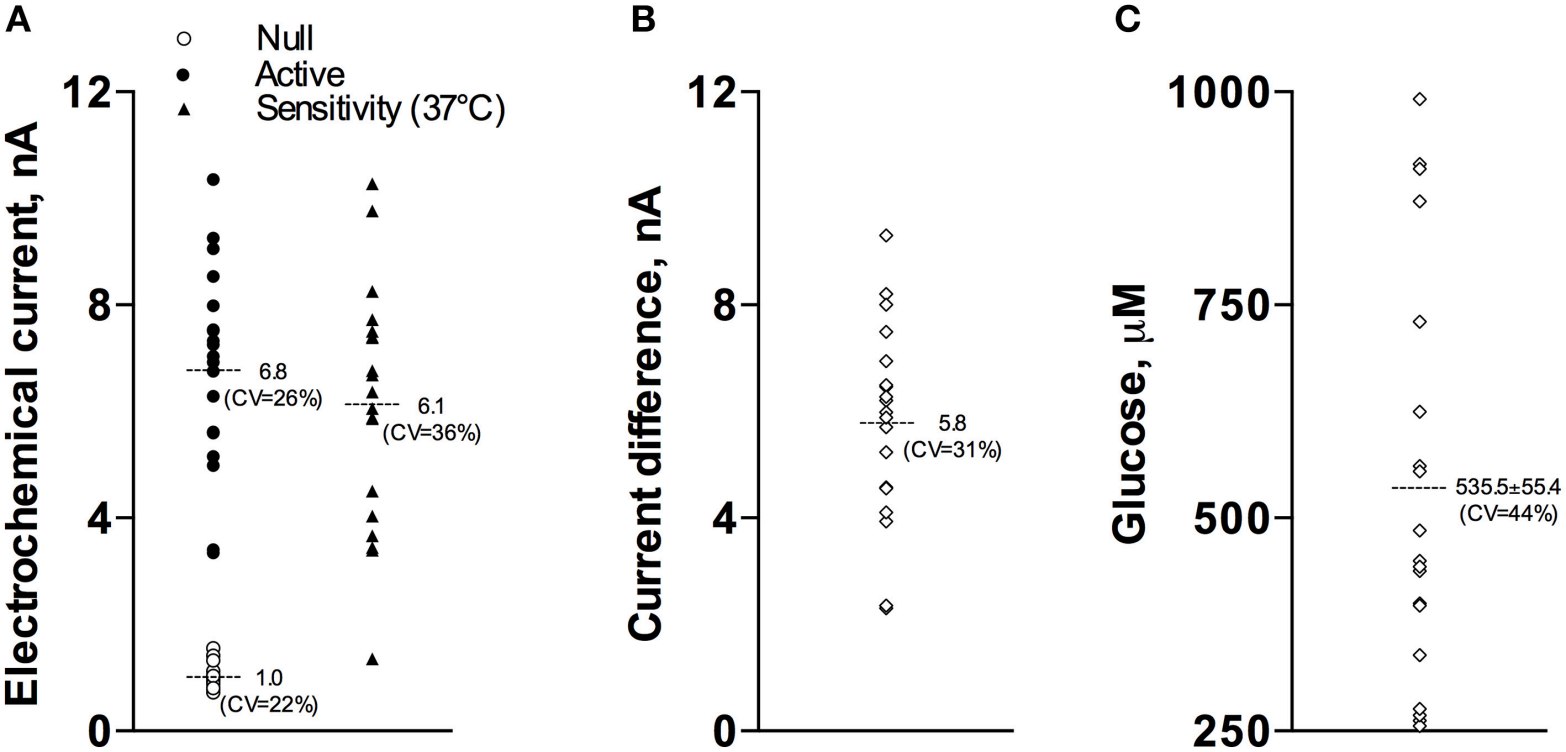
**Graphic representation of data used to determine basal concentrations of extracellular glucose in the NAc. (A)** shows the values of basal electrochemical currents detected by active (enzume-containing) and null (enzyme-free) sensors as well as the range of glucose sensitivity (nA/0.5 mM corrected for 37°C) of active sensors. **(B)** shows the range of differences between active and null currents detected at the same time points. **(C)** shows the values of basal glucose levels in this sample. See text for more detail explanations.

Our current analyses conducted for a relatively large data sample (17 active and 15 null recordings) revealed that the basal glucose values immediately before the injection of Equithesin were: 535.5 ± 55.4 μM (*SD* = 234.9 μM). Figure 1 shows how these basal glucose levels were determined. First, we measured basal currents detected by both glucose and null sensors immediately before the injection of an anesthetic drug (Figure 1A). Second, we determined the difference between each individual value of glucose current and the mean value of null current (Figure 1B). Third, we calibrated these differences in terms of concentration using the sensitivity values of each sensor adjusted to 37°C (Figures 1A,C).

**Figure 2:**
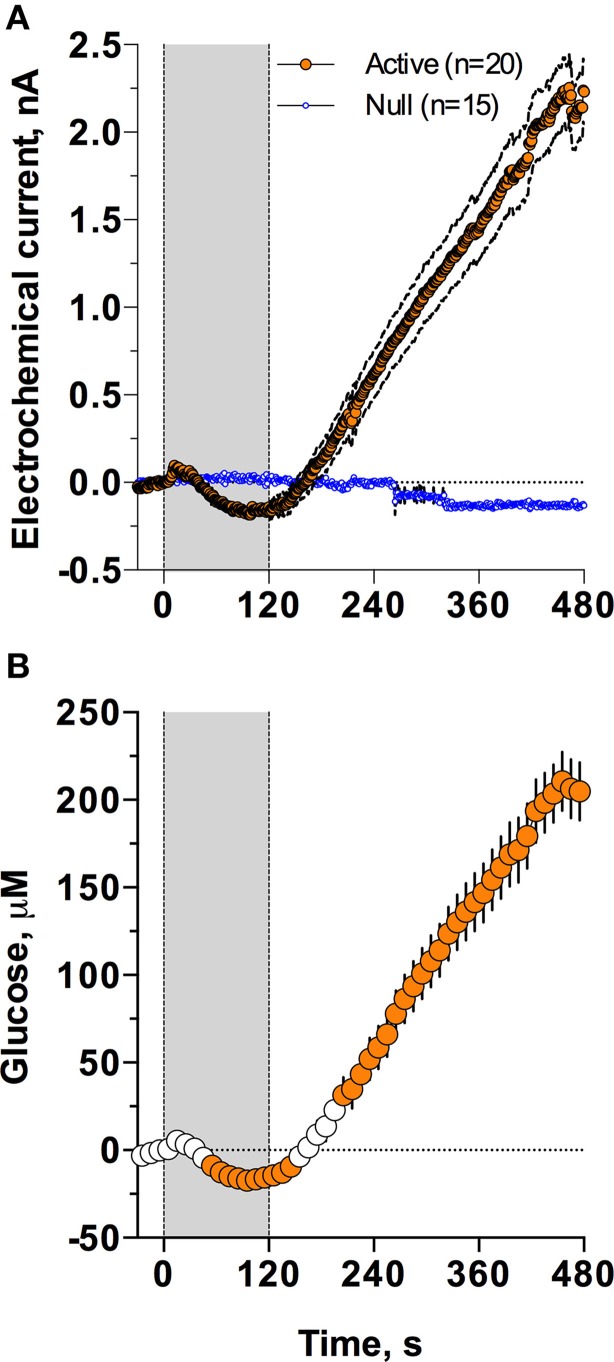
**Mean (± SEM) changes in NAc electrochemical currents (A; orange: glucose and blue: null sensors) and glucose concentration (B) induced by iv injection of Equithesin**. Gray area shows the duration of iv injection (0–120 s). Filled symbols in **(B)** show values significantly different from the pre-injection baseline (*p* < 0.05).

As shown in Figure 2, injection of Equithesin induces a bidirectional change in glucose currents with no changes in null current (Figure 2A). After subtracting the values detected by null sensors and calibrating the difference in terms of concentration (Figure 2B), we found that glucose levels slightly decreased during the injection but gradually and strongly increased from ~150 s, reaching ~210 μM at the final analysis point (8 min after injection onset). While one-way RM ANOVA applied to the entire analysis interval revealed a significant increase [effect of time: *F*_(48, 912)_ = 90.87; *p* < 0.001], the same analysis using a sliding time window revealed that initially glucose significantly decreased [*F*_(15, 285)_ = 7.71; *p* < 0.01], falling below baseline from 55 to 145 s after the injection onset.

### The time-course of NAc glucose elevation during general anesthesia

In Experiment II (six rats with active and two with null sensors), we examined the time-course of the NAc glucose responses induced by iv injection of Equithesin. Since two factors: a relatively large volume of the injection and the cool temperature of the injected volume could possibly contribute to certain components of the glucose response, we tested the effects of saline delivered at the same volumes and speed at room temperature, and the effects of Equithesin delivered at room (23°C) and body (37°C) temperatures. In this experiment, before drug or saline injections, we also examined NAc glucose response induced by an arousing stimulus; these data will be shown separately in the last part of the Results Section.

**Figure 3:**
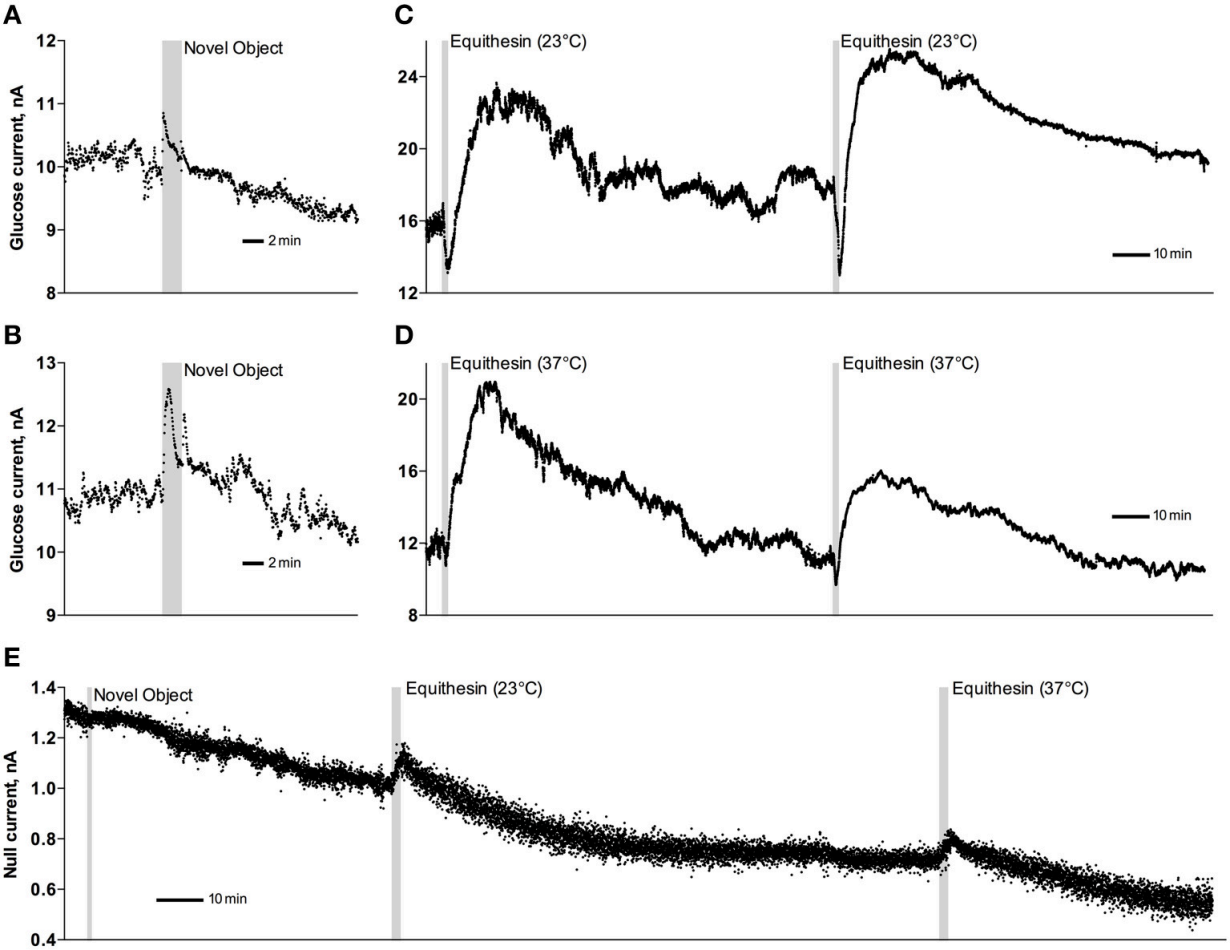
**Original examples of changes in electrochemical currents detected by glucose (A–D) and null (E) sensors following novelty test (A,B,E) and iv injections of Equithesin (C–E)**. Data were obtained with 1-s time resolution. Gray areas in each graph show the duration of stimulation or injection. Anesthetic drug was delivered at 23 and 37°C.

**Figure 4:**
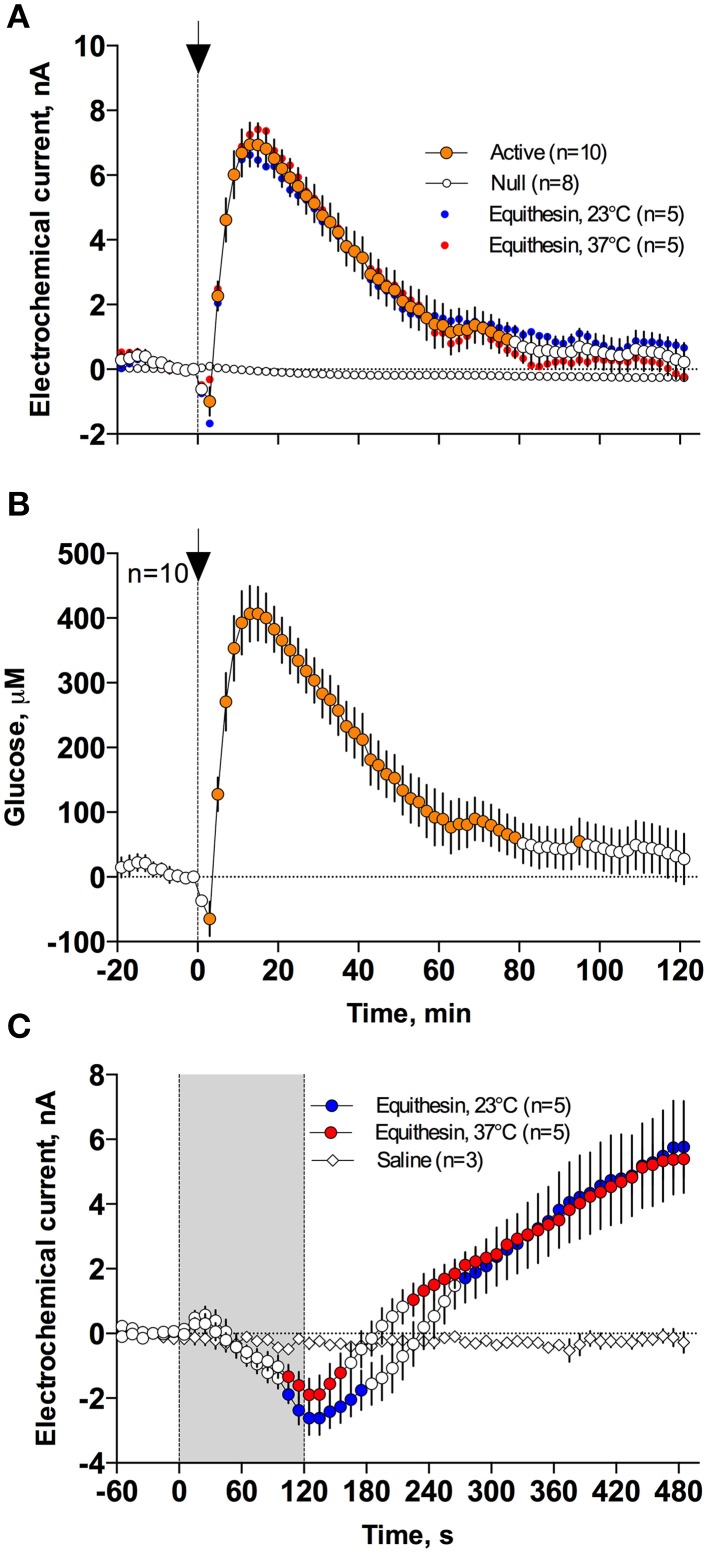
**Mean (± SEM) changes in NAc electrochemical currents (A,C) and resulting change in glucose concentration (B) after iv injection of Equithesin analyzed with slow (2-min bin) and rapid (10-s bin) time resolution. (A,C)** show changes detected by active (enzyme-containing) and null (enzyme-free) sensors for 120 min **(A)** and 8 min **(C)** after drug administration. Blue and red values show changes with the drug delivered at room (23°C) and body (37°C) temperatures, respectively. Filled symbols in each graph indicate values significantly different from the pre-injection baseline. N is the number of averaged tests.

As can be seen in Figure 3, an injection of anesthetic drug at both temperatures induced robust and prolonged increases in NAc glucose currents (Figures 3C,D) with no changes seen in null currents (Figure 3E). When analyzed statistically for the entire drug group (Figure 4A; *n* = 10 with 5 tests at 23°C and 5 tests at 37°C), the effect of time was highly significant for glucose current [*F*_(9, 549)_ = 47.66; *p* < 0.001] and not significant for null current. The mean of the null currents was then subtracted from individual glucose currents, and these values were calibrated to concentration values based on sensors' sensitivity corrected for brain temperature. We found that NAc glucose levels, after the initial weak drop during the injection (0–4 min), rapidly increased for ~10 min, peaked at 406 μM above baseline (range 172–608 or 720–1556 μM in terms of absolute concentration), and slowly decreased toward baseline (Figure 4B). In this data sample, the mean basal concentration of NAc glucose was 707.8 ± 44.7 μM (*SD* = 134.2 μM) and the mean drug-induced increase was about 60% above baseline.

While the increase was the predominant effect of general anesthesia, glucose currents initially decreased during the first 4 min after the injection onset (Figures 4A,B). This initial effect was clearly evident and significant when data were analyzed at a high temporal resolution [Figure 4C, 10-s bins; *F*_(49, 196)_ = 18.99 and 37.50 for cold and warm solutions, respectively; both *p* < 0.001]. This effect, however, was absent after saline injection at the same volume delivered at room temperature, suggesting its pharmacological nature. Although this inhibiting effect was stronger when Equithesin was delivered at room temperature (see Figures 3C,D), group analysis revealed no significant differences between the groups (Figure 4C). No differences were also seen in the slow time-course analysis; mean glucose currents in two groups (23 and 37°C) were superimposable within the entire drug effect.

### Brain and body hypothermia during general anesthesia and its underlying mechanisms

**Figure 5:**
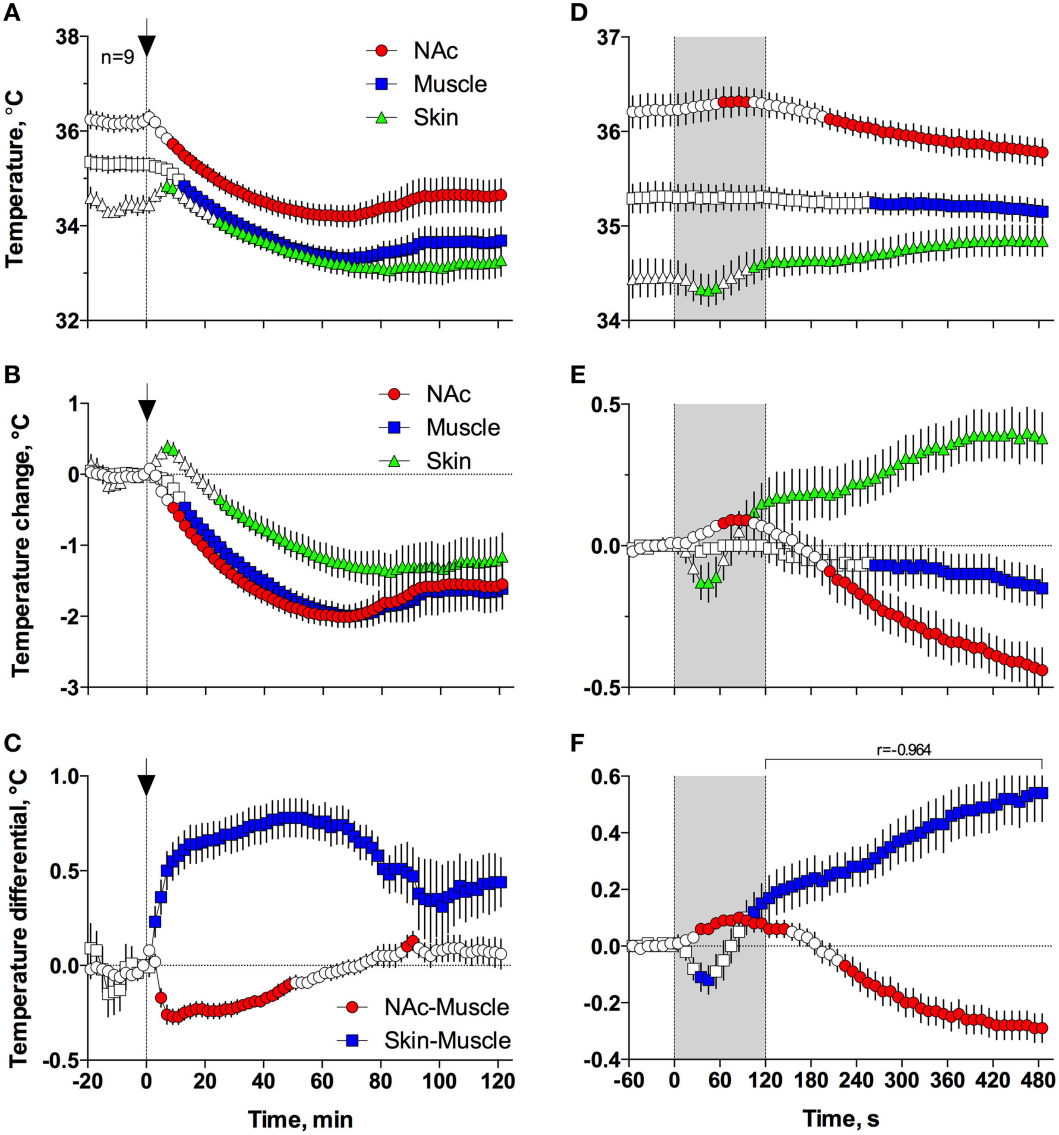
**Changes in different temperature parameters induced by iv injection of Equithesin, shown with slow (A–C; 2-min bin), and rapid (D–F; 10-s bins) time resolutions**. The top graphs **(A,D)** show absolute changes in NAc, temporal muscle, and skin temperatures; middle graphs **(B,E)** show relative changes in these parameters; and bottom graphs **(C,F)** show changes in NAc-Muscle and Skin-Muscle temperature differentials. Values significantly different from pre-injection baseline are shown as filled symbols. In **(A–C)**, the moment of injection is shown by a vertical hatched line (0 min) with an arrow; in **(D–F)**, duration of injection (0–120 s) is shown as gray shaded areas. In **(F)**, *r* is coefficient of correlation determined from 120 to 480 s post injection onset.

Figures 5A,B demonstrates that general anesthesia induced by iv injection of Equithesin induces robust and prolonged temperature decreases in the NAc, temporal muscle, and skin [effect of time; one-way RM ANOVA *F*_(61, 488)_ = 12.44, 19.57, and 19.50, respectively; all *p* < 0.001]. When analyzed at a slow time resolution (2-min bins), the decrease was strongest in the brain site (~1.8°C), slightly smaller in the muscle (~1.7), and minimal in the skin (~1.2°C). While generally correlative, temperature dynamics had important differences in each recording location. Temperature decreases in the brain were more rapid and stronger than in temporal muscle, resulting in a rapid decrease in the NAc-Muscle differential [Figure 5C; *F*_(61, 488)_ = 13.96; *p* < 0.001], suggesting decreased intra-brain heat production due to metabolic brain inhibition. This effect appeared at 5–6 min, peaked at 10–12 min, and slowly disappeared within 40–60 min post-injection. In contrast, due to the initial increase in skin temperature (first 10 min post-injection) coupled with a decrease in muscle temperature, the Skin-Muscle differential (a valid measure of peripheral vascular tone) rapidly increased from 2–4 min, stabilized at high levels (~0.7°C) at 20–80 min, and slowly decreased toward baseline, not reaching it at the final time point of analysis [effect of time; *F*_(61, 488)_ = 9.24; *p* < 0.001]. Therefore, both metabolic inhibition and strong skin vasodilation contribute jointly to anesthesia-induced brain and body hypothermia.

Since the most robust changes in temperature parameters occurred immediately after injection, the same parameters were analyzed at a high, 10-s temporal resolution for the initial 8 min after the injection onset (Figures 5D–F). Following this analysis, we found that NAc temperature initially increased and skin temperature transiently decreased immediately after the start of the injection but then, at ~60 s, both curves inverted and skin temperature began to increase and brain temperature began to decrease (Figures 5D,E). Importantly, the increase in Skin-Muscle differential, suggesting vasodilation, occurred earlier, during the injection. Conversely, the decrease in NAc-Muscle differential, suggesting decreased intra-brain heat production, occurred with a longer latency (~220 s; Figure 5F). Similar to the subsequent opposite effects, decreases in the Skin-Muscle temperature differentials occur much quicker (20-30 s latency) and they are more transient (~50 s) than the more tonic increases in the NAc-Muscle differential. Importantly, changes in NAc-Muscle and Skin-Muscle differentials correlated tightly (*r* = −0.964) from the onset of drug injection (Figure 5F). Therefore, the injection of Equithesin results in transient and immediate brain activation and vasoconstriction, followed by opposite effects that occur for a longer period of time.

### Relationships between anesthesia-induced changes in NAc glucose and temperature parameters

To understand the possible mechanisms underlying anesthesia-induced fluctuations in NAc glucose, we next examined how these changes correlate dynamically with NAc-Muscle and Skin-Muscle temperature differentials, which show, respectively, drug-induced changes in metabolic activity and the tone of skin vessels.

**Figure 6:**
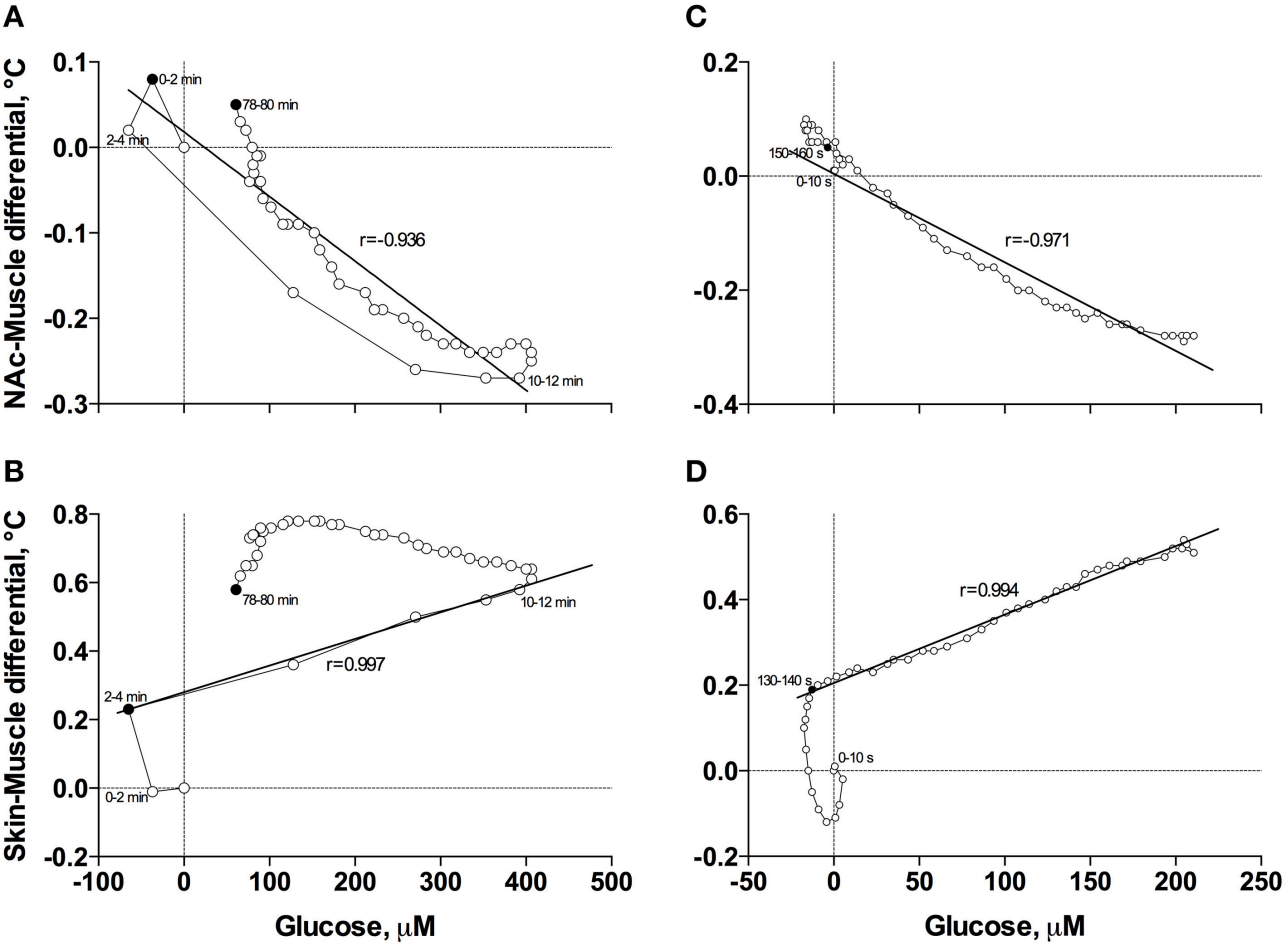
**Correlative relationships between anesthesia-induced changes in NAc glucose and two primary indices reflecting metabolic activity (NAc-Muscle differential; A,C) and vascular tone (Skin-Muscle differential; B,D) analyzed with slow (A,B) and rapid (C,D) time resolutions**. For explanations see the text.

When the data were analyzed at a slow time-resolution (2-min bin) for the entire period of a significant glucose change (0–80 min), the increases in glucose correlated with decreases in NAc-Muscle differentials (Figure 6A). The correlation appeared from ~3 min post-injection, was strong within the entire duration of glucose elevation (*r* = −0.936; *p* < 0.001) and close to linear. After glucose peaked and descended toward baseline, the correlation was maintained but became slightly weaker and less linear. Even stronger correlation between these parameters (*r* = −0.971, *p* < 0.001) was evident when data were analyzed with high temporal resolution for the initial 8 min post-injection (Figure 6C).

In contrast to the inverse correlation with an index of metabolic activity, glucose levels directly correlated with increases in Skin-Muscle differentials (Figure 6B). This correlation was evident and exceptionally strong from 3 to 12 min post-injection (*r* = 0.997, *p* < 0.001), when glucose levels gradually increased, but disappeared after glucose peaked and descended to baseline. The high linearity and exceptionally strong correlation between these parameters (*r* = 0.994, *p* < 0.001) was especially evident when data were analyzed with high temporal resolution for the first 8 min post-injection (Figure 6D).

### Temperature and glucose responses induced by arousing stimulation

**Figure 7:**
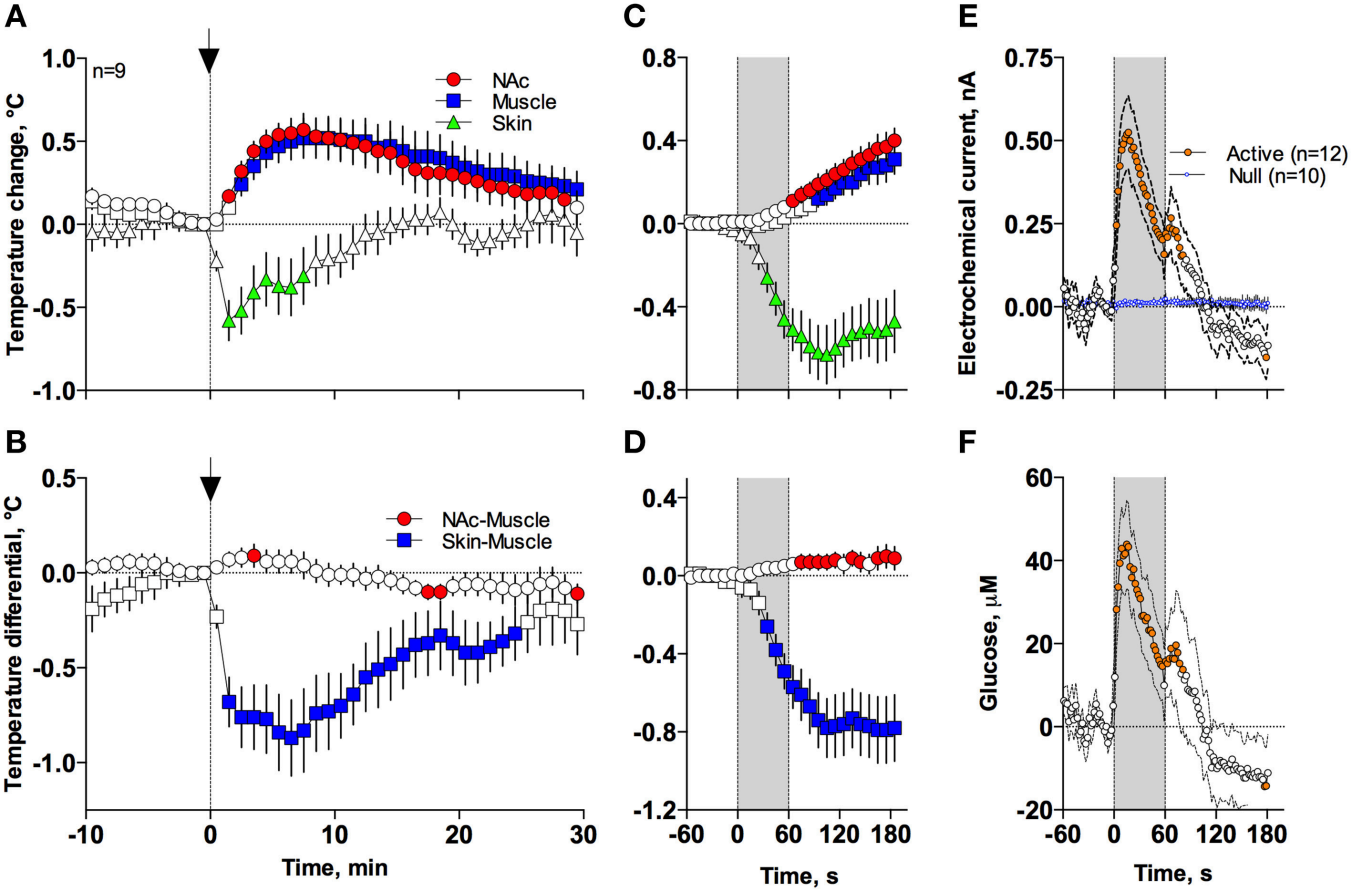
**Mean (± SEM) changes in temperatures (A–D), NAc electrochemical currents (E; orange: glucose and blue: null sensors), and glucose concentration (F) induced by the novelty test**. The rat at time 0 was introduced to a glass beaker, which was removed from the cage at time 60 s. In **(A,B)**, onset of arousing stimulation is shown as vertical hatched line with arrow and in **(C–F)**, duration of stimulation (0–60 s) is shown as a gray shaded area. Filled symbols in each graph show values significantly different from baseline.

In contrast to profound hypothermia during anesthesia, arousing sensory stimulation (presentation and removal of a glass beaker) slightly increased brain and muscle temperatures and decreased skin temperatures [Figure 7A; *F*_(51, 408)_ = 20.40, 10.98, and 2.67, respectively; all *p* < 0.01]. Sensory stimulation also induced significant but opposite changes in NAc-Muscle and Skin-Muscle differentials [*F*_(51, 408)_ = 5.02 and 5.41, respectively]. Due to more rapid and stronger increases in brain temperature, the NAc-Muscle differential transiently increased, suggesting enhanced heat production due to metabolic activation. In contrast, the Skin-Muscle differential rapidly and strongly decreased, suggesting vasoconstriction (Figure 7B). This effect was rapid (significant at 2 min after beaker presentation), peaking at 3–7 min before slowly returning to baseline.

These differences in speed and intensity of temperature responses in different recording locations were more evident following rapid time-course analyses (Figures 7C,D). In this case, skin temperature showed a significant decrease at the fourth data point (or 30–40 s from stimulation onset), while increases in brain and muscle temperatures became significant at 60–70 and 90–100 s after stimulus onset, respectively. Similar differences in timing and response magnitude were also evident for NAc-Muscle and Skin-Muscle differentials.

While NAc glucose also increased during novelty test, the changes were different from those seen during anesthesia (see original examples Figures 3A,B). Using high time-resolution analysis (2-s bins), we found that glucose currents significantly increased [*F*_(91, 1001)_ = 15.05; *p* < 0.001], while null currents show no changes (Figure 7E). Transformation of these current changes into concentration values revealed that glucose levels rapidly rise, becoming significant from the second data point (2–4 s) following beaker presentation (Figure 7F). These increases peaked at ~15 s and decreased toward baseline, showing an additional small peak after beaker removal. In contrast to the high-magnitude glucose rise seen during the development of anesthesia, this phasic increase was relatively small in magnitude (~42 μM or 5–10% above baseline). This pattern of the NAc glucose response differed significantly from the dynamics of temperature parameters, which showed much slower but longer changes.

## Discussion

Our present study revealed that NAc extracellular glucose levels tonically increase during the development of general anesthesia. These increases tightly correlate with increases in Skin-Muscle temperature differentials, suggesting that anesthesia-induced brain hyperglycemia may result from a massive inflow of glucose from the cerebral vessels due to their vasodilation. This robust pharmacological effect drastically differs from both the slow, gradient-dependent glucose increases after consumption of glucose-containing products and rapid, neural activity-regulated glucose increases induced by arousing stimuli and occurring during behaviors and it could explain the existing controversy regarding basal glucose levels in different animal preparations.

### What are the basal levels of extracellular glucose in brain tissue?

Extracellular glucose could be measured by using microdialysis (Fellows et al., 1992; Fray et al., 1997; McNay and Gold, 1999; McNay et al., 2001), providing an accurate estimate of its basal levels and tonic fluctuations. However, this technique is essentially slow and does not allow for the detection of rapid fluctuations in glucose levels. This goal could be achieved by using glucose oxidase-based biosensors coupled with amperometry (Hu and Wilson, 1997; Wilson and Gifford, 2005)—an alternative approach for assessing second-scale fluctuations in extracellular glucose levels (see for review Kiyatkin and Wakabayashi, 2015). While this approach is usually not instrumental for quantifying basal levels of neurochemicals, the parallel use of glucose-null sensors, which are equally sensitive as glucose sensors to multiple physical and chemical contributors, allowed us to estimate extracellular glucose levels under physiologically relevant conditions. Our previous (540–700 μM; Kiyatkin and Lenoir, 2012; Wakabayashi et al., 2015) and current (540–707 μM) estimates for the NAc are in line with previous zero-net-flux microdialysis (470–1000 μM Fellows et al., 1992; Fray et al., 1997; McNay and Gold, 1999; McNay et al., 2001) and electrochemical estimates (350–600 μM (Lowry and Fillenz, 1997; Netchiporouk et al., 2001; Kealy et al., 2013) obtained in different brain structures in awake, freely moving rats. However, these values are much lower than classic calculated values (2–4 mM; Siesjo, 1978; Lund-Andersen, 1979) and both electrochemical and microdialysis estimates obtained in anesthetized animal preparations [2.4 mM for ventromedial hypothalamus; Silver and Erecinska, 1994; 3.3 mM for cortex in Ronne-Engström et al. (1995); 2.6 mM for hippocampus in Hu and Wilson (1997)]. Although our present measurements were limited to the ventral striatum and basal glucose levels could vary in different brain structures, this study clearly shows that general anesthesia tonically increases extracellular glucose well above the levels seen under normal physiological conditions.

### Physiological fluctuations in NAc glucose and their mechanisms

Glucose used for brain metabolism arrives from the arterial blood, where its concentration is maintained at much higher and relatively stable levels. Due to this concentration gradient, glucose continuously enters the brain's environment by facilitated diffusion via the GLUT-1 glucose transporter (Duelli and Kuschinsky, 2001). Due to the rise in this concentration gradient, brain glucose levels substantially increase after consumption of glucose-containing products (Wakabayashi and Kiyatkin, 2015a) and systemic or intra-gastric glucose administration (Dash et al., 2013). In addition to this “passive,” gradient-dependent mechanism, glucose levels could transiently increase or decrease in different brain structures due to changes in neuronal activity, which directly affect the tone of cerebral vessels and local cerebral blood flow. As shown in this study with a novelty test (see Figures 3, 4), NAc glucose rapidly rises and peaks within seconds after the presentation of a novel object and quickly returns to baseline, showing a small second peak after the novel object's removal. Importantly, glucose rises well before and much faster than increases in NAc-Muscle temperature differential, suggesting rapid neural regulation of this response that precedes the increases in intra-brain heat production due to metabolic brain activation (see Figure 7). Therefore, due to the efficient neuro-vascular coupling, the brain is able to receive energetic substances (glucose and possibly oxygen) for its metabolic activity well in advance of any actual demand, thus preventing possible metabolic deficit. In contrast to “passive” gradient-dependent glucose entry into brain tissue, which has been shown to similarly affect different brain structures, neural activity-triggered fluctuations in glucose are structure-specific, as shown previously by comparing the ventral striatum and *substantia nigra pars reticulata* (Kiyatkin and Lenoir, 2012). Most neurons in the NAc have slow, sporadic discharges, and show phasic excitations to sensory arousing stimuli (Kiyatkin and Rebec, 1996, 1999), and glucose levels are phasically increased by these stimuli. Conversely, glucose levels phasically decrease in *pars reticulata*, where most neurons have high activity rate and show phasic inhibitions following sensory stimulation (Schultz, 1986; Windels and Kiyatkin, 2004).

### Anesthesia-induced brain hyperglycemia and its mechanisms

The robust tonic rise in NAc glucose following the development of general anesthesia could represent another mechanism of glucose entry into the brain extracellular environment. An exceptionally strong correlation between this glucose response and an index of vasodilation could suggest that this effect is mediated by anesthesia-induced cerebral vasodilation, which enhances glucose entry even with minimal changes or even decreases in local cerebral flow typical of anesthesia (Mishra, 2002). In this study we examined the NAc glucose response induced by an iv-delivered mixture of pentobarbital and chloral hydrate, but increases in extracellular glucose have been previously reported with different anesthetic drugs (i.e., pentobarbital, chloral hydrate, ketamine+xylazine, isoflurane), in different brain structures, and by using both microdialysis (Fellows et al., 1992; Canal et al., 2005) and electrochemical detection (Osborne et al., 1997; Lowry et al., 1998; Netchiporouk et al., 2001; Dash et al., 2013; Kealy et al., 2013). Although the magnitude of these increases varied for different drugs in different studies, the consistency of this effect may suggest that brain hyperglycemia occurs during the development of general anesthesia independently of the type of anesthetic drug. Importantly, glucose levels strongly increased during anesthesia induced by pentobarbital (Osborne et al., 1997; Canal et al., 2005; our unpublished data), a known inhibitor of metabolic brain activity that induces robust brain and body hypothermia (Kiyatkin and Brown, 2005).

Similar to the large-magnitude effects found in previous studies, our quantitative analysis revealed that the NAc glucose increase induced by Equithesin is strong, exceeding 60% of quiet-resting baseline and moving absolute glucose levels in this structure to 720–1556 μM. High-magnitude effect of anesthesia is also indirectly supported by data obtained in anesthetized animal preparations, which revealed unusually high “basal” levels of extracellular glucose (2.4–3.3 mM, Silver and Erecinska, 1994; Ronne-Engström et al., 1995; Hu and Wilson, 1997). Despite the consistency of this phenomenon, its underlying mechanisms remained a matter of speculation.

To explore the possible mechanisms of this phenomenon, our glucose recordings were supplemented by high-speed monitoring of brain, muscle, and skin temperatures. While anesthesia-induced decreases in brain and body temperature is a well-known phenomenon (Lenhardt, 2010), the three-point recording paradigm used in this study allowed us to assess two basic mechanisms (intra-brain heat production due to metabolic brain activation and changes in vascular tone) underlying the hypothermic effects of general anesthesia. By using correlation and regression analyses, we found that the ascending phase of NAc glucose rise tightly correlates with increases in Skin-Muscle differentials, which indicate peripheral vasodilation. This correlation appeared ~150 s after the start of 120-s injection and was exceptionally strong (*r* =>0.99) and linear for the entire ascending phase of the glucose increase. Since the tone of cerebral vessels is the primary factor affecting glucose entry into brain tissue, this rise could reflect the enhanced glucose entry from the arterial blood due to dilation of cerebral vessels. While the direct data on the vascular response in the NAc are absent, vasodilation confirmed in this study at the level of peripheral vessels could also occur at the level of cerebral vessels (ter Laan et al., 2013). Although, it has been previously speculated that anesthesia-induced brain glucose rise could be dependent upon the rise in blood glucose levels, direct measurements during pentobarbital and ketamine-xylazine anesthesia revealed that increases in blood glucose are incomparably weaker and, most importantly, much slower than those seen in the brain (Canal et al., 2005). Furthermore, as shown in our previous study employing iv injections of glucose (Wakabayashi et al., 2016), increases in NAc glucose are relatively small after transient doubling (~15%) or even tripling (~35%) of blood glucose levels.

While extracellular glucose could phasically rise due to local neural activation and subsequent vasodilation and this mechanism was confirmed here in our novelty test (see above), it is unlikely that this factor is involved in mediation of brain hyperglycemic response because most anesthetic drugs, including pentobarbital and chloral hydrate, are known to inhibit the activity of ventral striatal neurons (West, 1998; Chen et al., 2001; Windels and Kiyatkin, 2006). Finally, it is unlikely that drug-induced metabolic inhibition *per se* could be a mechanism determining the strong rise in glucose seen in this study. Although our thermorecording data suggest that Equithesin anesthesia decreases brain metabolic activity, this effect develops with some latency and is weaker than vasodilation. However, glucose changes strongly and negatively correlated with decreases in the NAc-Muscle differential (*r* => 0.97), suggesting that glucose rise is related to metabolic inhibition. While it is possible to speculate that the diminished glucose uptake by brain cells (i.e., metabolic inhibition) could shift the balance of extracellular glucose levels to their increase, it is unclear how this cellular change could account for the robust rise in glucose during the development of general anesthesia. A possible explanation could arise when we look at the relationship between anesthesia-induced metabolic inhibition and vasodilation, which also tightly correlated (*r* = −0.96) from ~2 min post drug injection (see Figure 5), suggesting interrelatedness between these two parameters and their corresponding physiological mechanisms. Therefore, both metabolic brain inhibition and vasodilation could be two different but related effects induced by central actions of anesthetic drugs.

### Local action of the anesthetic drugs during the injection

In addition to the primary effect of anesthesia, high-speed analysis of electrochemical signals revealed that the NAc glucose rise is preceded by a transient decrease in NAc glucose during the injection (see Figures 3, 4). As confirmed with our control tests using equal-volume saline injections and different temperatures of the injected drug, this effect has a pharmacological origin. While further studies are necessary to clarify the mechanisms underlying this transient effect, it appears that it is triggered by the local action of the injected drug on the afferents of sensory nerves that densely innervate the internal walls of veins and the heart atrium (Lee et al., 2005). This visceral sensory stimulation creates an ascending neural signal that is rapidly transmitted to the CNS and induces a transient vasoconstriction that could determine a transient drop in glucose levels. In support of this mechanism, a transient vasoconstriction was observed during high-resolution analysis of our thermorecording data (see Figures 5D–F). It is important to note that this transient effect could not be detected with slower measurement techniques (i.e., microdialysis) and it even almost disappears with a slow time resolution analysis of the electrochemical data.

### Functional implications

The two primary mechanisms that determine glucose entry into brain tissue (i.e., via a “passive” rise in blood-brain gradient and “active” neuro-vascular coupling) could explain changes in extracellular glucose occurring under normal physiological conditions. As shown in this study, another mechanism involving metabolic brain inhibition and vasodilation is engaged during general anesthesia, resulting in strong brain hyperglycemia. While in this study our measurements were limited to the NAc, an area with high-density of GLUT-1 transporters (Zeller et al., 1997), evidence from existing literature suggests that this phenomenon could occur to different extent in other brain structures, thus explaining the existing controversy regarding basal glucose levels in different animal preparations. The powerful effects of general anesthetics on brain metabolic activity, the tone of peripheral and central vessels, and the entry of glucose into the extracellular domain also question the validity of physiological evaluations of these processes conducted in anesthetized animal preparations. While the direct relationships between metabolic brain activation, increased cerebral blood flow, and entry of glucose and oxygen into brain tissue generally hold under normal physiological conditions, these relationships become much more complex and can alter drastically following exposure to pharmacological drugs that have distinct effects on these physiological processes.

## Author contributions

Concept and design: EK; Research performance: RB and EK; Data analyses: RB and EK; Drafting the manuscript: EK and RB. The initial data included in Experiment I were obtained with Ken T. Wakabayashi, who was involved in early discussions regarding the concept and implications of this study.

## Funding

Supported by the National Institute on Drug Abuse - Intramural Research Program, NIH (1ZIADA000566-05).

### Conflict of interest statement

The authors declare that the research was conducted in the absence of any commercial or financial relationships that could be construed as a potential conflict of interest.
